# Exploring the journey of Responsible Business Model Innovation in Asian companies: A review and future research agenda

**DOI:** 10.1007/s10490-022-09813-0

**Published:** 2022-03-22

**Authors:** Domitilla Magni, Rosa Palladino, Armando Papa, Patrice Cailleba

**Affiliations:** 1 Department of Economics and Management, University of Catania, Catania, Italy; 2 Department of Economics, Management and Statistics (DEMS), University of Milano-Bicocca, Milan, Italy; 3 Faculty of Communication Sciences, University of Teramo, Teramo, Italy; 4 HSE University, Moscow, Russian Federation; 5 PSB Paris School of Business, PSB Reseach lab, Paris, France

**Keywords:** Responsible Innovation Journey, Business Model Innovation, Asia, Business Ethics, Organizational Capabilities, Knowledge Exchange

## Abstract

Responsible Business Model Innovation is increasingly becoming a relevant challenge in academic research and business practice, mainly in the Asian context. Changes in business models are widely acknowledged as a key strategy for achieving long-term innovation. However, little is known about the design journey of Responsible Business Model Innovation. By applying the knowledge-based view and stakeholder theory, this paper introduces the founding pillars of Responsible Business Model Innovation, namely: Corporate Social Responsiveness, Inclusiveness, and Reflective Knowledge Exchange. Based on the analysis of extended bodies of literature published between 2011 and 2021 on business model innovation, sustainability innovation, stakeholder theory and responsible innovation, the article explores the state of the art of business ethics and dynamic capabilities in Asian organizations. Findings show that recent research in the field of sustainability and ethical values are improving the impact on business models, thus encouraging the advent of Responsible Business Model Innovation. This article contributes to the emerging field of responsible innovation and offers novel theoretical and practical implications for academy and practitioners, including a first attempt to develop a road map to be followed to achieve of sustainable and ethical values for business and society at large.

Business Model Innovation is an exploratory process mediated by “opportunity recognition and entrepreneurial bricolage” (Guo et al., 2016: 533). Although the literature considers the disruptive role of Business Models (BM) (Abdulkader et al., 2020; Langley et al., 2021; Malhotra & Van Alstyne, 2014; Teece, 2010; Zott & Amit, 2010), the academic research contribution on innovation in BM design and content needs further exploration. Despite this, Business Model Innovation (BMI) was still under-investigated by scholars after the beginning of the new century (Teece, 2006; Chesbrough, 2007). This has generated a theoretical void, which research on BMI has subsequently tried to fill (Fruhwirth et al., 2020; Schneider & Spieth, 2013), especially regarding strategy (Spieth et al., 2019; Verhoeven & Johnson, 2017), resilience (Ramdani et al., 2020), and business cycle models (Laudien & Daxböck, 2017). Recently, academia named this new research stream ‘Responsible BMI’ (RBMI). A pioneering basis for RBMI research was laid out by scholars on eco-innovation (Sarkar, 2014; Wickson & Carew, 2014) and clean technologies (Baldassarre et al., 2017; Shahzad et al., 2020). One of the first-ever comprehensive views on sustainability and responsible transaction in academic research is provided by Kern et al. (2019), noticing that policy mix conceptualizations have been included in policy-makers’ considerations. This paper offers a holistic perspective of RBMI that includes responsiveness, inclusiveness, and knowledge exchange dimensions. Starting from the point that BMI theory still lags in relation to Corporate Social Responsiveness (CSRV) (Beliveau et al., 1994; Lee, 2007; van Staden & Hooks, 2007), this paper systematically includes environmental (Sarkar, 2014), health (Downs & Velamuri, 2016), economic, and social (Murray et al., 2017; Vladimirova, 2016 & 2019) concerns as part of the challenges raised by BMI.

In addition, the paper aims to examine the application of RBMI in the Asian socio-economic business context. Previous literature has already argued for the agility and flexibility of Asian companies (Witt & Redding, 2014; Paulet & Rowley, 2017; Hasegawa & Witt, 2019), highlighting that the latter are drivers for sustainable change and consequently potential promoters of RBMI. Overall, Asian entrepreneurs seem now more concerned by sustainability goals rather than mere profitability objectives: indeed, scholars and practitioners have focused on the Asian context to investigate the drivers that trigger sustainable change in the digital era. Specifically, the literature has focused on China (Birkin et al., 2009; Murray et al., 2017), India (Chaudhuri et al., 2018; Jose, 2008; Trehan & Sinha, 2020), Indonesia (Danarahmanto et al., 2020) and Thailand (Ketprapakorn & Kantabutra, 2019), by confirming the findings of previous research on responsible and sustainable challenges (Masurel, 2007; Rodgers, 2010; Thongpoon, 2012). This trend has been reinforced by the emergence of resource scarcity (in terms of Information Technologies, Human Resources, or commodities) which can be an advantage for innovation whether it be in the middle-east (Ramdani et al., 2020), in Iran (Moradi et al., 2021), in China (Chen et al., 2019; Guo et al., 2016; Ji et al., 2016; Loon & Chik, 2019), and in ASEAN countries as a whole (de Sousa Jabbour et al., 2020; Magni et al., 2021). RBMI can consequently constitute a key success factor for business development in the digital age (Chua et al., 2020) and in a post covid-19 world (Zahra, 2021) within a region that is still considered to be the factory of the world (Harun & Zainol, 2018; Permatasari & Dhewanto, 2013).

Drawing on an in-depth review of the literature which extends recent research (Downs & Velamuri, 2016; Fruhwirth et al., 2020), this paper aims to identify the main drivers suggested by the literature (Bocken et al., 2013 & 2014; DaSilva, 2018; Geissdoerfer et al., 2018; Hope, 2018; Raith & Siebold, 2018) for building an architecture of RBMI in Asian business organizations (Guo et al., 2016; Chin et al., 2021; Hossain, 2021).

Based on the relevant theme of CSRV, we introduce the founding pillars of RBMI to discuss how emerging concepts in the field of sustainable business models (Bocken et al., 2013; Hope, 2018; Vladimirova, 2016; Yang et al., 2017) allowed organizations to address the challenges of Corporate Social Responsibility (CSR) implementation and corporate ethics. To address our objectives, we apply the knowledge-based view of a firm (Grant, 1996) and stakeholder theory (Freeman, 1984; Freeman & Phillips, 2002; Freeman et al., 2010) to understanding the role of stakeholders in inclusiveness and business ethics. Moreover, recent research (Bridoux & Stoelhorst, 2016; Jones et al., 2016) places an emphasis on the joint-value creation favoured by stakeholder theory. Putting aside a traditional “transactional” approach (self-interest and financial incentives) vs. “relational” approach (compassion, honesty, integrity, and kindness) dichotomy, we show how recent scientific developments have favoured knowledge exchange between stakeholders as envisioned by practitioners (DaSilva, 2018; Vladimirova, 2019) and scholars (Loon & Chik, 2019). Doing so, we make a theoretical contribution to recent research (Nardi, 2021), which analysed companies’ choices to engage in substantive, instead of merely symbolic CSR.

Based on the work of Stilgoe et al. (2013) on responsible innovation, we deepen the discussion on the nature of joint value creation as part of a theoretical game framework (Swierstra & Jelsma, 2006). We show how social exchange theory (Begley et al., 2010) applied to business (Chen & Choi, 2005) paved the way towards innovation with consumers (i.e., stakeholders)—from democratic (Von Hippel, 2006) to free innovation (Von Hippel, 2016)—and then to RBMI.

Finally, we present how RBMI can reinforce Asian business competitiveness by identifying antecedents and forecasting in Asian industry, thus providing useful managerial and academic implications by filling current gaps in the literature that deals with the topic. The rest of the article is structured as follows: Section 2 provides the theoretical backgrounds of CSRV, stakeholder theory, and reflective knowledge exchange by representing them as the pillars of RBMI. Section 3 describes the methodology used in this study; Section 4 explores the bibliometric analysis of the results; findings are discussed in Section 5; Section 6 focuses on the conclusion, managerial implications, limitations of the study, and future research directions.

## Theoretical framework

### Corporate social responsiveness and responsible business model innovation

A firm’s environmental capability is a part of its strategic capabilities, and they are critical to the success of the organizations’ environmental initiatives and to reducing its ecological footprint (Qi et al., 2020). Environmental pollution has increased in recent decades, and many regulatory authorities’ policy makers and stakeholders are putting growing pressure on businesses to implement green process innovations which comprise clean technology solutions (Khan et al., 2021c; Singh, Del Giudice, et al., 2021).

Nowadays, firms are promoting new values within their business environment, integrating ethical values and sustainability into their business model to enhance their own CSRV. The effectiveness of the implementation of CSRV within organizations is a relevant challenge for society at large (Jones et al., 2016; van Staden & Hooks, 2007). CSRV refers to the way an individual or an organization responds to a social need (Stilgoe et al., 2013). In line with new research streams and along with economic and environmental sustainability, CSRV emphasizes the role of corporate ethical values and the importance of maintaining clear and open dialogue with stakeholders to foster ethical and socially responsible strategy for new social challenges (Genus & Stirling, 2018; Golob & Bartlett, 2007).

It is now understood that CSRV is part of the RBMI development process. Khan et al. (2021b), consider that institutional pressures, and environmental and structural changes in a market influence the environmental strategies of managers, but also feed into the implications for regulators and policymakers to find common ground on issues of CSR, environmental, social, and economic sustainability. Indeed, socially sustainable firms are purposely committed to affirming green and ethical values within the wider business environment (Shahzad et al., 2020). In this way, CSRV expresses the integration of ethical concerns into a firms’ strategic vision, with organizations effectively managing the challenges of social and ethical impact within their business environment and through stakeholders (Malhotra et al., 2017). The efficient implementation of CSRV helps firms to gain economic advantage (Bai & Chang, 2015). Organizations willing to adopt these responsive approaches can evaluate and meet the economic, environmental, and social expectations of all stakeholders, thus empowering effectiveness on relationships with employees and with consumers (Akram et al., 2019; Zhu et al., 2014).

This context creates the need to critically analyse the topic of sustainability challenges, sustainable innovation, and responsible innovation in terms of RBMI. Bhatt et al. (2020) identify the predominant focus on the development of firms’ capability for environmental management. In addition, by applying the lens of environmental management, several studies have examined the impact of lean tools on firms’ sustainability processes (King & Lenox, 2001). While focusing on responsible innovation, Stilgoe et al. (2013), have already discussed the relevance of innovation within the organization’s routines and processes by summing up the four integrated dimensions of responsible innovation, as follows: anticipation, reflexivity, inclusion, and responsiveness.

In this vein, several studies have highlighted the importance of moving from a “merely symbolic” CSRV to a “substantive” approach, thus engaging the formulation of RBMI for sustainable business performance (Carayannis et al., 2015; Nardi, 2021). This shifting paradigm has been trigged by a continuous demand for awareness of new stimuli reducing the negative socio-environmental externalities associated with the firm’s production output (Nardi, 2021). Thus, an explicit formulation of CSRV within RBMI supports organizations in addressing responsible transformation in socioeconomic settings for “long-term survival” (Caputo et al., 2021; Chesbrough, 2007). In general, RBMI is founded on the concept of Responsible Innovation (RI), defined as the continuing interests of inclusive approach innovation towards various environments, whether social, cultural, economic, or environmental (Stilgoe et al., 2013). RI refers to four main dimensions: (i) inclusion, i.e., engaging various stakeholders (Malhotra et al., 2017); (ii) anticipation, i.e., understanding future opportunities for the wider business environment and society (Wickson & Carew, 2014); (iii) responsiveness, i.e., providing transparency and accessibility to innovative results and social needs (Stilgoe et al., 2013); and (iv) reflexivity, i.e., circular knowledge within the organization (Chen & Choi, 2005). These CSRV and RI pillars enable organizations to implement strategic RBMI to align social and ethical values within the business environment and the wider context.

### Inclusiveness and business ethics as the basis of a responsible stakeholder approach

Firms’ social responsiveness is based, in part, on the ability to meet the needs of stakeholders (Lee, 2007). Based on Freeman’s seminal theory (Freeman, 1984; Freeman & Phillips, 2002; Freeman et al., 2010), it is relevant to consider the overall inclusiveness of the stakeholder theory. The stakeholder theory encompasses most business theories to provide sustainable competitive advantage (Jones et al., 2018). Several studies advocate the stakeholder theory, which derives from systemic theories, i.e., the “open systems”, which currently characterize the debates between the organization and its environment (Bridoux & Stoelhorst, 2016; Halme & Korpela, 2014; Hammann et al., 2009). This highlights the simultaneous possibility of action and reaction between the firm and the environment, mostly referring to the complex interactions between the behaviour of the various third parties involved, such as stakeholders (Singh et al. 2020). It is necessary to meet the expectations of stakeholders and third parties, whose behaviour can influence the success of the firm. ‘Relational’ resources, even if not intended as inputs into business processes, act upon and determine competitive advantage over competitors (Singh et al., 2019). This approach implies that the organization’s success coincides with meeting the expectations of all third parties involved: therefore, firms must not only operate with the aim of maximizing profit, but also with the intention of realizing the interests of all stakeholders (Litz, 1996). This has strengthened the already existing interrelationship between stakeholder theory, CSRV, and business ethics (Riege & Lindsay, 2006; Singh et al., 2019). The stakeholder theory has been established as the main perspective for the analysis of social responsibilities. The achievement of sustainable development is an objective often found in management decisions, which are the result of a meticulous analysis of the demands coming from supra-systems, with the intention of orienting organization choices towards their expectations and aims (Cillo et al., 2019; Owen et al., 2009). By following this perspective and based on pioneering papers (Bridoux & Stoelhorst, 2016; Jones et al., 2018; Schaltegger et al., 2012), our conceptual framework goes beyond the traditional stakeholder approach. Indeed, it presents an exhaustive view of stakeholder theory targeting CSRV and RBMI, mainly based on joint-value creation: the relevant shifting from “transactional” to “relational” approach to create responsible and shared value for all stakeholders, by respecting principles of inclusiveness and social welfare (Bridoux & Stoelhorst, 2016).

More recently, green innovation capability development has been recognized as a context for collaborative networks (Evans et al., 2017; Papa et al., 2020; Singh, Del Giudice, et al., 2021). BMI and sustainability innovations require the rethinking of several business aspects, such as stakeholder relationships. Evans et al. (2017) have developed a unified theoretical perspective for understanding the way BMI leads to better organizational economic processes thanks to the collaborative stakeholders’ network.

Through the RBMI lens, our paper intends to provide a comprehensive and inclusive framework for shareholder theory through the increasing stakeholder contribution built around communal sharing, equality matching and balanced reciprocity, all based on the relational model theory and opening the way to free innovation (Carayannis et al., 2014; Schaltegger et al., 2012; Von Hippel, 2016).

### From “stakeholder knowledge view” to reflective knowledge exchange

The notion of “responsible management” for the benefit of the environment has led organizations to invest in developing social auditing techniques, stakeholder relations models, social audits, as well as codes and practices with responsible and inclusive systems of corporate governance (Genus & Stirling, 2018; Simmons, 2004). The firm can be considered as a system involving all stakeholders and, therefore, the focus is on the network of relationships between the different actors, both internal and external, which may have an impact on the dynamics of the whole organization (Chen & Choi, 2005). The firm and its operations are constantly subject to a continuous and responsible process of knowledge exchange within the business environment, representing CSRV as conscious knowledge and experience processes (Gangi et al., 2019; Papa et al., 2020). Reflective knowledge exchange (RKE) is defined as a metaphor of network collaboration into the business environment (Jones, 2015). Knowledge-intensive sectors encourage their organizations to share knowledge and stimulate its accumulation and combination within open innovation ecosystems (Bereznoy et al., 2021; Keszey, 2018). The innovative lens of social exchange theory (Begley et al., 2010) applied to business (Chen & Choi, 2005) opened the way to link innovation between firms and consumers. According to Von Hippel (2006, 2016) and by applying the open innovation lens, a firm’s strategy is triggered by consumers and stakeholders to achieve democratic and free innovation. RKE and open innovation allow firms to manage organizational change in the value co-creation process (Abdulkader et al., 2020; Bogers et al., 2017). Research on innovation management, RKE, and dynamics capability have observed that organizations are willing to join social and ethical value among stakeholders (Santoro et al., 2019). Indeed, an interesting perspective on RKE considers that dynamics capabilities such as critical resources offer competitive advantage, thus generating innovative solutions through appropriate knowledge exchange practices (Nguyen et al., 2015; Singh, Mittal, et al., 2019). On the one hand, sedimentation of new knowledge is based on the comparison with past experiences and investments (Sedighi et al., 2018); on the other hand, assimilation is based on the characteristics of the knowledge or on those of the organization and dyadic alliances (Wang & Tarn, 2018). Moreover, the application of joint knowledge depends on technological opportunities (amount of relevant external knowledge) and their appropriateness (ability to protect innovation) (Segarra-Ciprés & Bou-Llusar, 2018; Santoro et al., 2019). Hence, high potential absorptive capacity translates into greater strategic flexibility due to the ability of the firm to reconfigure and renew its existing resources and competencies (Papa et al., 2020; Ramachandran, 2018). This is reflected into the RBMI, as realized absorptive capacity affects the improvement of the financial or productive performance of an organization through the combination of acquired and existing knowledge (Majchrzak et al., 2012). Furthermore, RKE optimizes the design of RBMI in several respects, i.e., a) it improves the relationship with all actors involved in strategic decisions, b) it triggers an omnichannel communication process for all stakeholders, and c) it implements the dynamic capacities of the companies allowing them to achieve the objectives of economic and social sustainability that they had set.

**Fig. 1:**
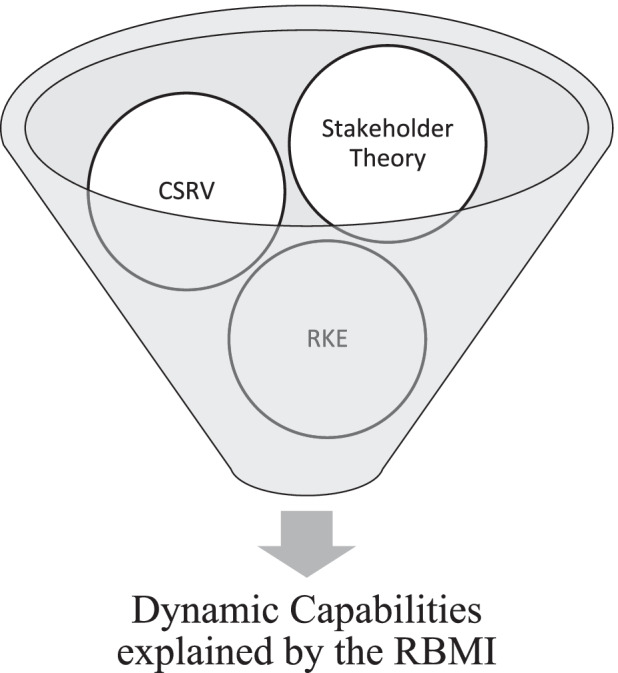
Conceptual framework. Source: Authors’ Elaboration

Figure 1 shows the conceptual framework used in our paper.

## Methodology

### Research protocol, database and search strings

Our methodology is based on a systematic literature review (SLR), observing an objective and replicable protocol (Tranfield et al., 2003). Due to the uncertainty surrounding the consequences and impacts of responsible innovation on the organizational skills of business ethics, the SLR makes it possible to map and evaluate all results available in the field, integrating the knowledge of academics and professionals and minimizing errors (Danese et al., 2018; Denyer & Tranfield, 2009; Khan et al., 2021b; Khanra et al., 2020a). The SLR provides a thematic classification of scientific contributions on topics of interest highlighting the areas most explored as well as the aspects that still need to be investigated and exploited (Tandon et al., 2020).

The research protocol envisaged the following order: 1. extrapolation of documents from databases, 2. manual selection of relevant documents, 3. identification of the most cited documents and 4. manual integration of other influential articles.

**Fig. 2:**
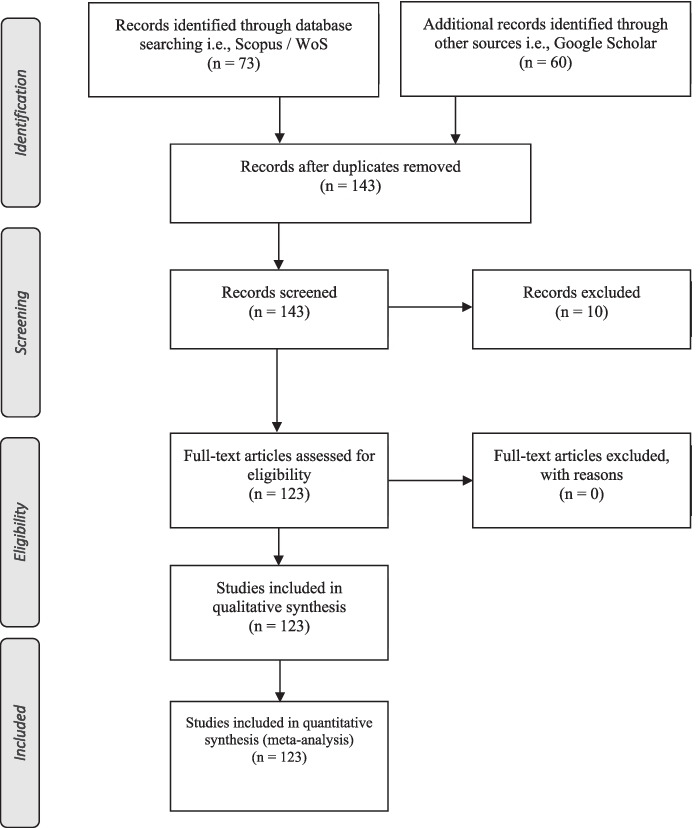
PRISMA *Flow Diagram*. Source: Authors’ Elaboration

Figure 2 contains the summary of the individual steps observed to identify and evaluate the documents for each phase of the research.

For the identification of relevant studies, the first phase of our methodology used two different databases, Web of Science (WoS) and Google Scholar (GS) (Kable et al., 2012). WoS is among the most popular databases in socio-economic research, because it can be associated with Scopus due to its volume (Hicks & Wang, 2011), but does not require data cleaning (Zupic & Čater, 2015). Furthermore, we integrated the analysis through a manual search in GS, considering the citation levels of further relevant articles published in high quality journals (Rashman et al., 2009).

The journals were selected based on the relevance of the content with respect to the topics investigated. We selected well-known journals to integrate publications on Innovation, Business Models and Business Ethics, such as *Journal of Knowledge Management*, *Journal of Business Research*, *Journal of Responsible Innovation* and *Technological Forecasting and Social Change* (Okoli & Schabram, 2010).

We searched for relevant articles from 1990 to 2021, using eight search strings:

[Business Model innovation] AND [Business Ethics *].

[Business Model innovation] AND [Corporate Social Responsiveness *].

[Business Model innovation] AND [Free Innovation].

[Business Model innovation] AND [Asia] OR [China] OR [India] OR [Indonesia] OR [South Korea] OR [Malaysia].

[Business Model innovation] AND [Knowledge Sharing].

[Business Model innovation] AND [Stakeholder * Inclusion*].

[Business Model innovation] AND [Reflexivity].

[Business Model innovation] AND [Responsible Innovation*].

The research criteria based on groups of words, using the asterisk where necessary to select the relevant documents, made it possible to broaden the search to all relevant contributions with respect to the topic, highlighting the correlation between the results reported for each string (Fink, 2019). Using “Business Model Innovation” as a key substrate of the research, we selected all the studies collected in the field of Business Ethics and Corporate Social Responsiveness, to identify the main drivers suggested by the literature to build a responsible innovation architecture in the Asian industry.

During the second phase, we filtered the relevant documents through content analysis, proceeding to read the title, keywords, and abstracts of each article, according to the protocol of our investigation. Through content analysis, we highlighted the relevance of each document, systematizing the information collected to highlight the link with the purpose of the research. In this phase, each author worked separately, after which the full set of results were compared, to guarantee their reliability (Krippendorff, 1980).

In the third phase of the research, to avoid neglecting further relevant contributions, we integrated a manual search in GS using the same search strings and observing the same time criterion.

Finally, in the last phase of the analysis, the authors studied each article analytically, identifying the relevant aspects with respect to the topic of investigation. After excluding inconsistent and duplicate documents, we put together a database of 123 articles.

To provide a qualitative-quantitative description of the results, our study conducted a bibliometric analysis of the selected articles, before exploring the discussion of the contents. The bibliometric analysis can be found in Section 4.

## Bibliometric analysis results

### Descriptive analysis

This section presents the descriptive results of the research. Following the guidelines of Donthu et al. (2021), we conducted a bibliometric analysis by reproducing scientific maps that highlight the correlation index between sources, authors, and documents in relation to the substrate of knowledge incorporated in the dataset. The collected articles were analysed in Bibliometrix (Aria & Cuccurullo, 2017), to reconstruct the global network of field research, highlighting the trends developed over time and space to trace the evolution of the topics.

**Fig. 3:**
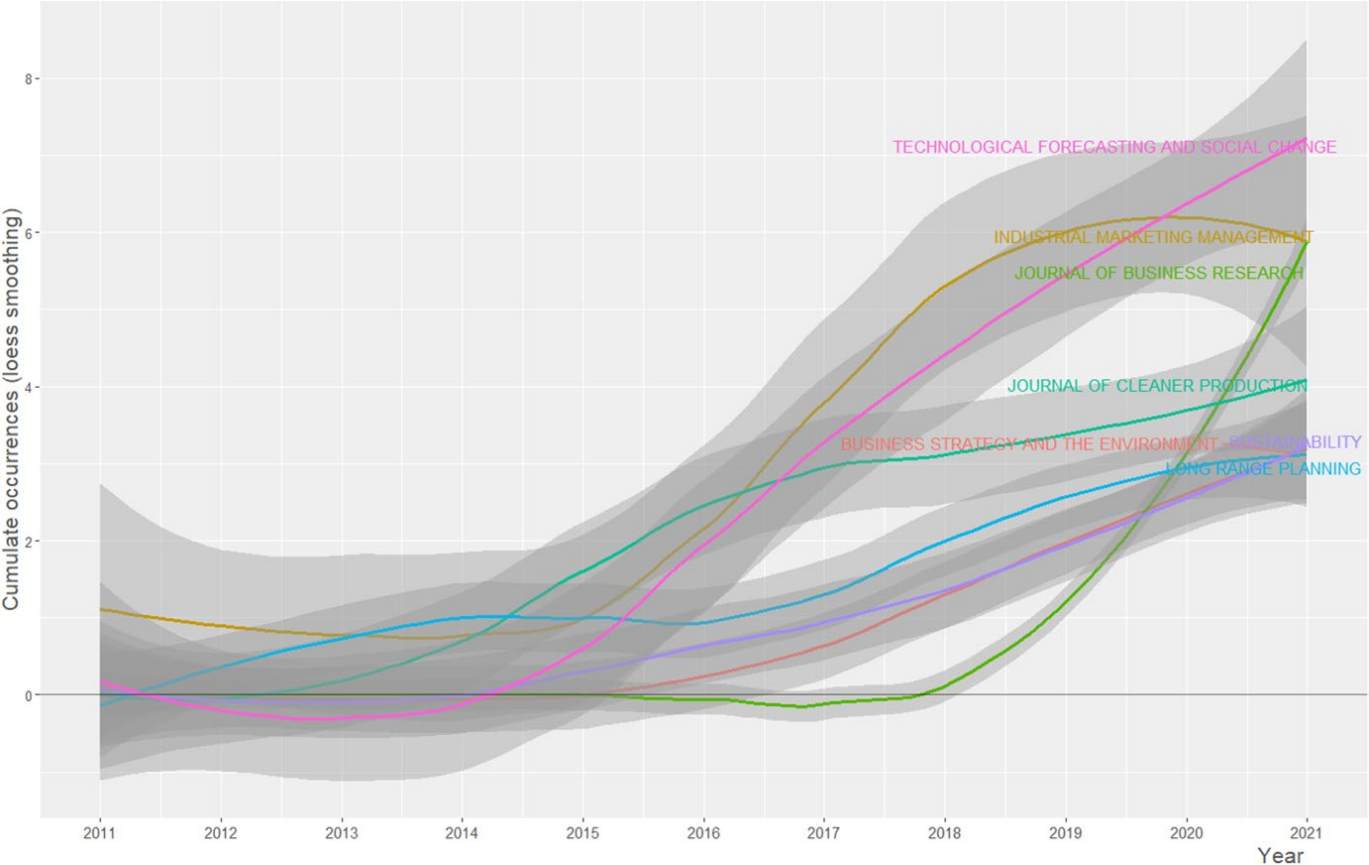
Source Growth. Source: Authors’ Elaboration

**Fig. 4:**
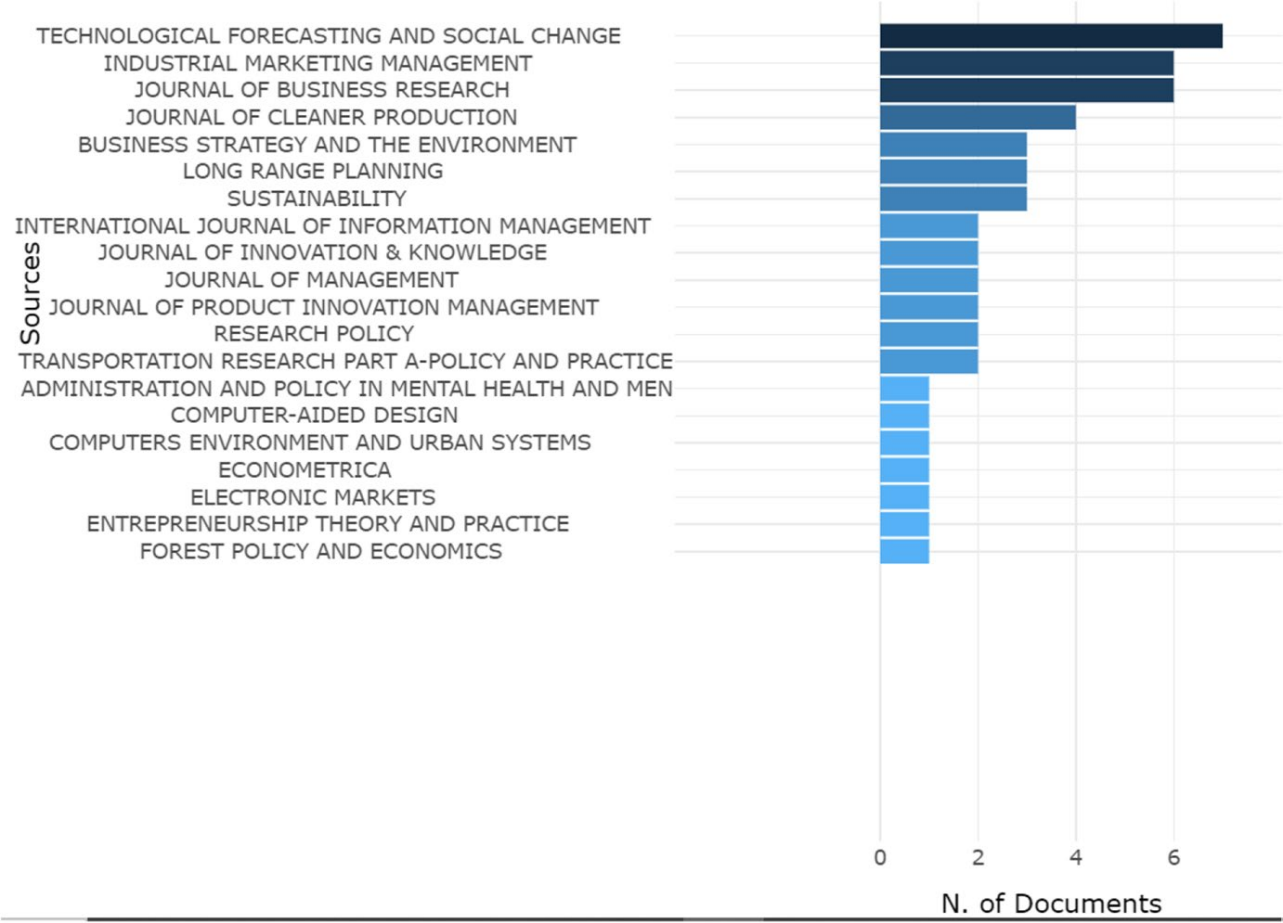
Most Relevant Sources. Source: Authors’ Elaboration

Bibliometric analysis is based on the citation index of scientific production on a topic to define the intellectual structure of a topic, trace its boundaries and limits (Khanra et al., 2020b). Furthermore, through cluster mapping, bibliometric analysis makes it possible to reach a holistic understanding (Tandon et al., 2021), exploring the possible relationships between related themes, opening new perspectives for analysis and new horizons for research on RBMI (Khan et al., 2021a). Through the evaluation of the impact factor and the citational indices of the articles, bibliometric analysis allows one to create a “transparent” and “reproducible” SLR (Aria & Cuccurullo, 2017: 959–960), based on the scientific mapping of information and the description of the conceptual data. From a first descriptive analysis of the database, the innovative nature of the topic emerges, which has been the subject of study since 2011, with an ever-increasing interest on the part of scholars. As Figs. 3 and 4 show, the journals that received the largest number of articles on this topic embrace research areas between management and business, including: *Technological Forecasting and Social Change, Industrial Marketing Management, Journal of Business Research and Journal of Cleaner Production*. Our bibliometric research covers the last 10 years: from 2011 to 2021.

**Fig. 5:**
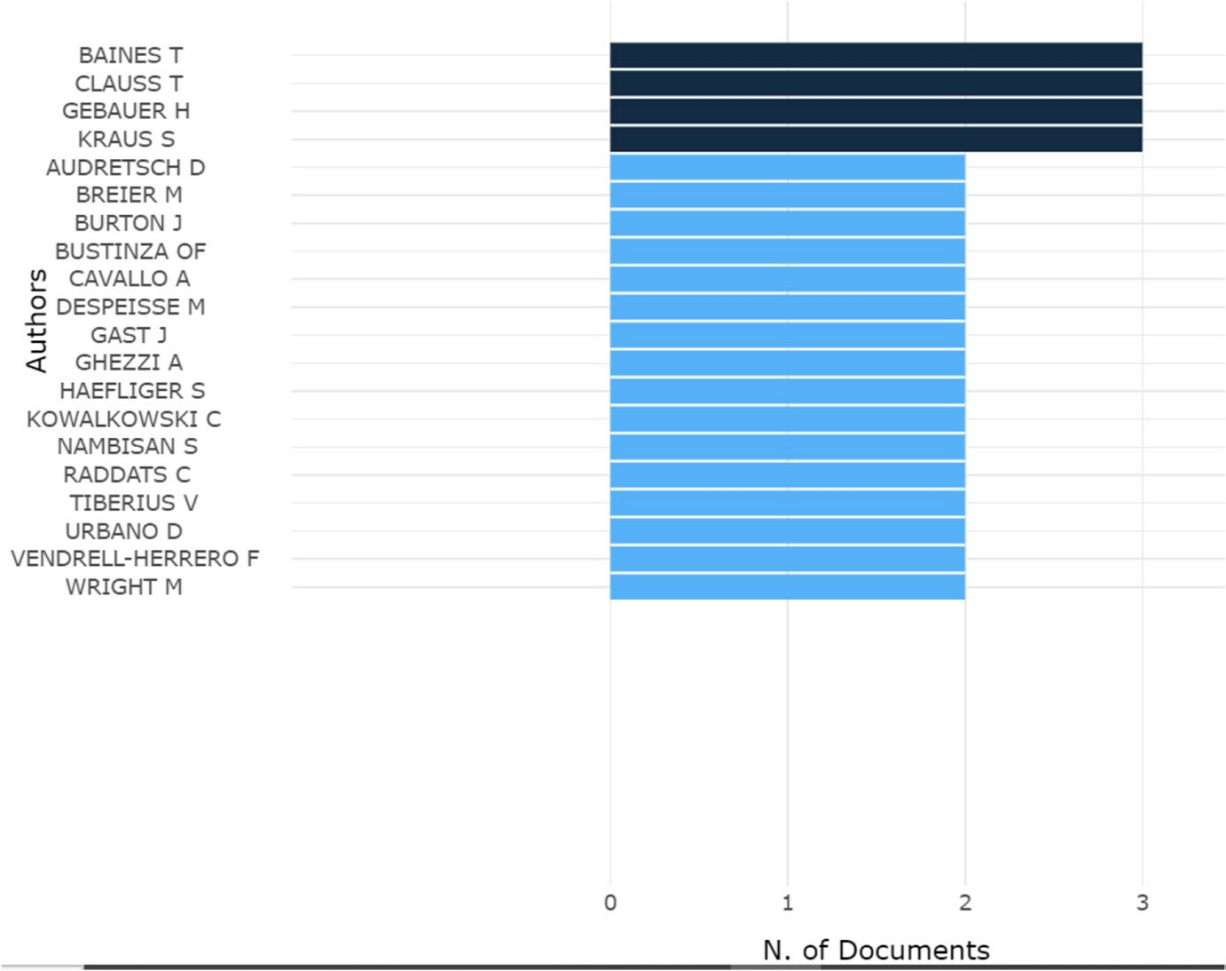
Most Relevant Authors. Source: Authors’ Elaboration

Furthermore, considering the output of authors, Fig. 5 shows the list of scholars who have contributed most to the study of the topic, counting for at least three articles in the database.

### Data analysis

**Fig. 6:**
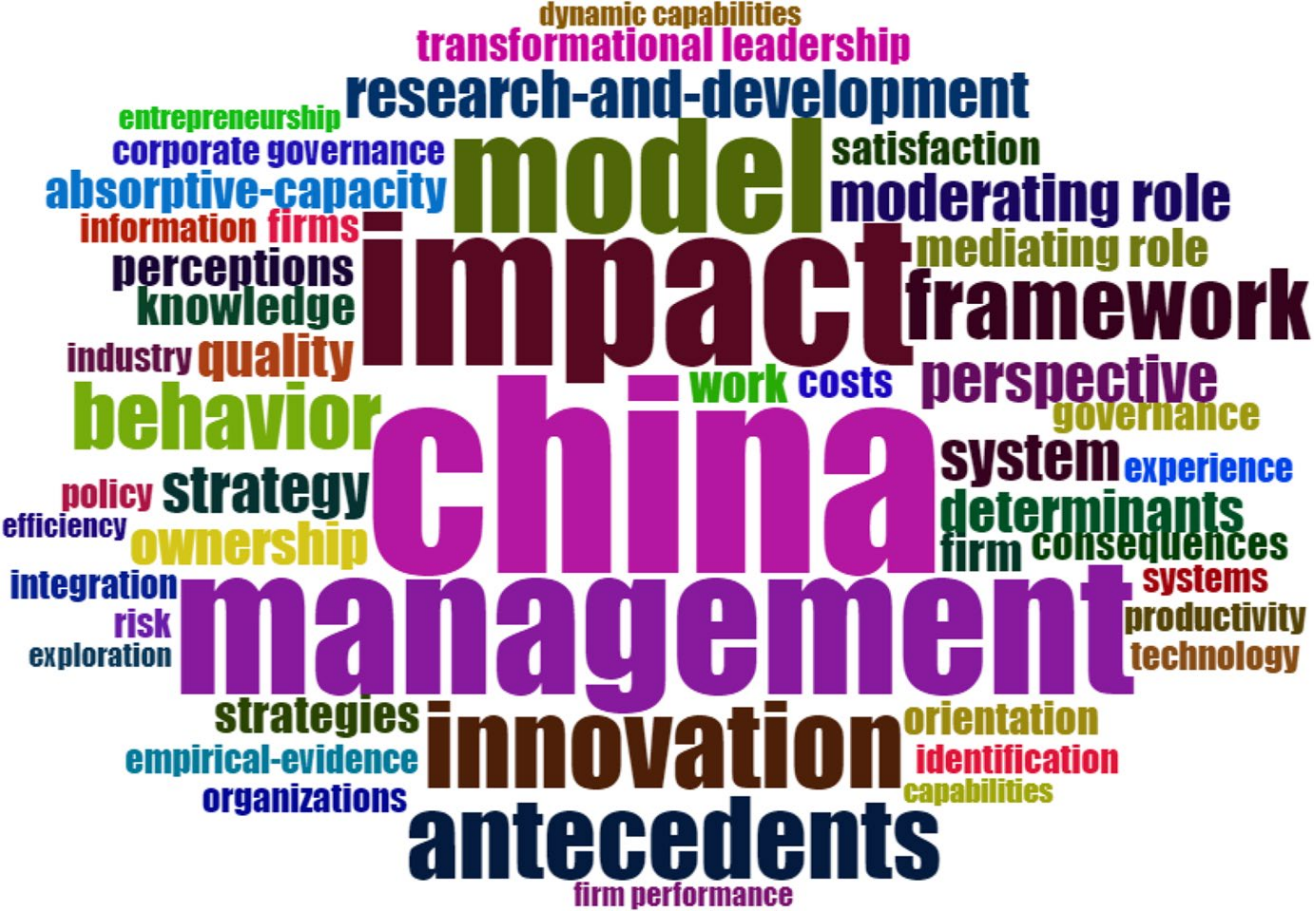
WordCloud of RBMI for the Asian context. Source: Authors’ Elaboration

Via the network extraction technique, based on the content of the documents present in the database, the analysis of the sample data was carried out (Aria & Cuccurullo, 2017). Figure 6 reports the co-word analysis, which shows the conceptual structure of the survey field, collecting the most common keywords in the data collection (Callon et al., 1983). The “WordCloud” map illustrates the cognitive basis of the database, reconstructing the similarity index between the documents based on the keywords (Fig. 6). The most common words in the collected articles will be of greater size and will be in the centre of the map. The major keyword of the analysis is “China”, which occurs 29 times in the data collection and is in the centre of the map. The words “impact”, “management”, “model”, “innovation” follow, which fill the field for the entire survey period (*ex multis* Breier et al., 2021; Langley et al., 2021; Broekhuizen et al., 2021; Ferasso et al., 2020; Geissinger et al., 2020; Kraus et al., 2020; Henry et al., 2020; Kohtamaki et al., 2019; De Massis et al., 2018; Ford & Despeisse, 2016).

The sequence described by the co-word analysis is particularly significant because it highlights the link between the topics investigated. Focusing our attention on the Asian context, the paper investigates the impact of innovation on business models, exploring the path towards value creation, through technologies, strategies and skills that orient entrepreneurship towards responsible organizational models.

**Fig. 7:**
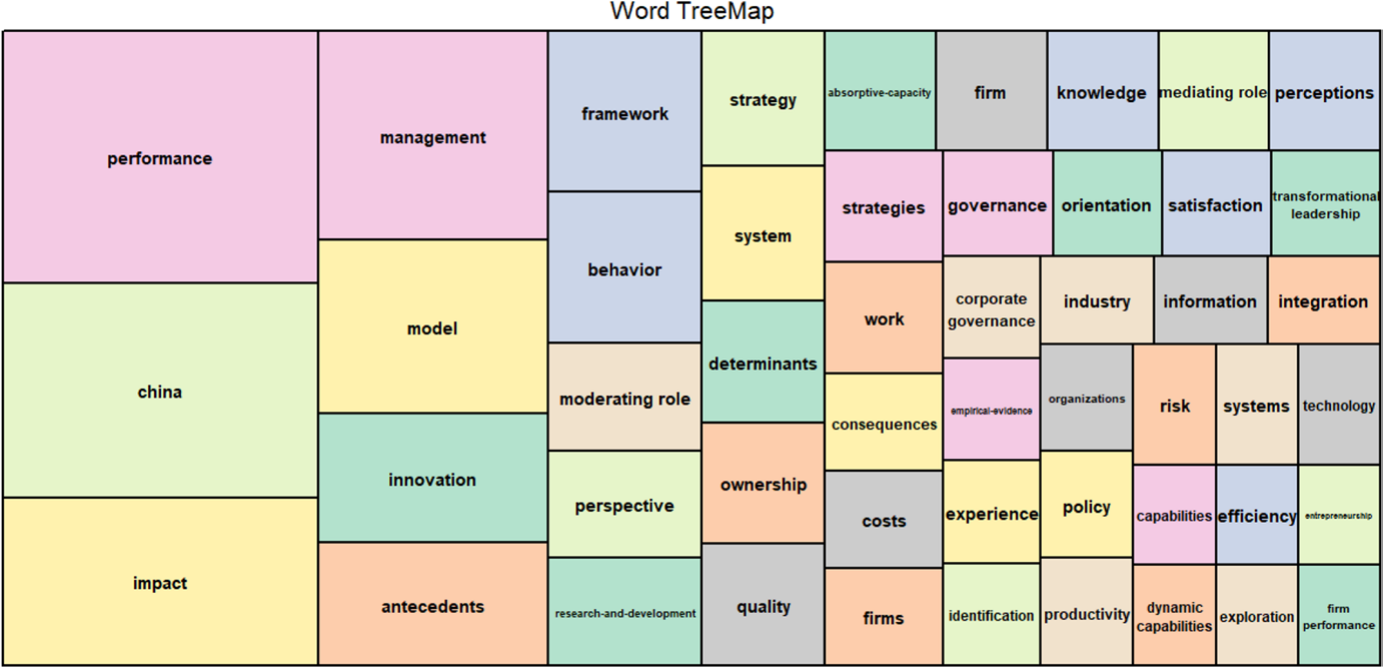
TreeMap. Source: Authors’ Elaboration

Based on the analysis of the conceptual structure, Fig. 7 reports the TreeMap of the main topic included in the dataset, following a hierarchical order (Shneiderman & Plaisant, 1998). The most frequently used keywords are boxed in rectangles of various sizes that provide a dynamic overview of the concepts, to trace the evolution of the topics. The size of the rectangle refers to the information structures most used in the sample. Colour matching expresses the creation of pairs on emerging themes represented by smaller rectangles.

The larger rectangles contain the words “performance”, “China” and “impact” which express the macro-themes of our investigation. The theme of performance is linked by colour to the emerging research sectors, which come from the occurrence index of the words in the database. The words “management”, “strategies”, “governance” and “capabilities” have connections with performance. Therefore, the words “perspective”, “strategy”, and “entrepreneurship” are related to China. This co-occurrence index confirms that the strategic choices of business organizations are correlated to the organizational capabilities of companies, helping to define the architecture attracting responsible innovation and spreading CSRV practices among the Asian industry (Gold et al., 2001; González-Masip, et al., 2019).

**Fig. 8:**
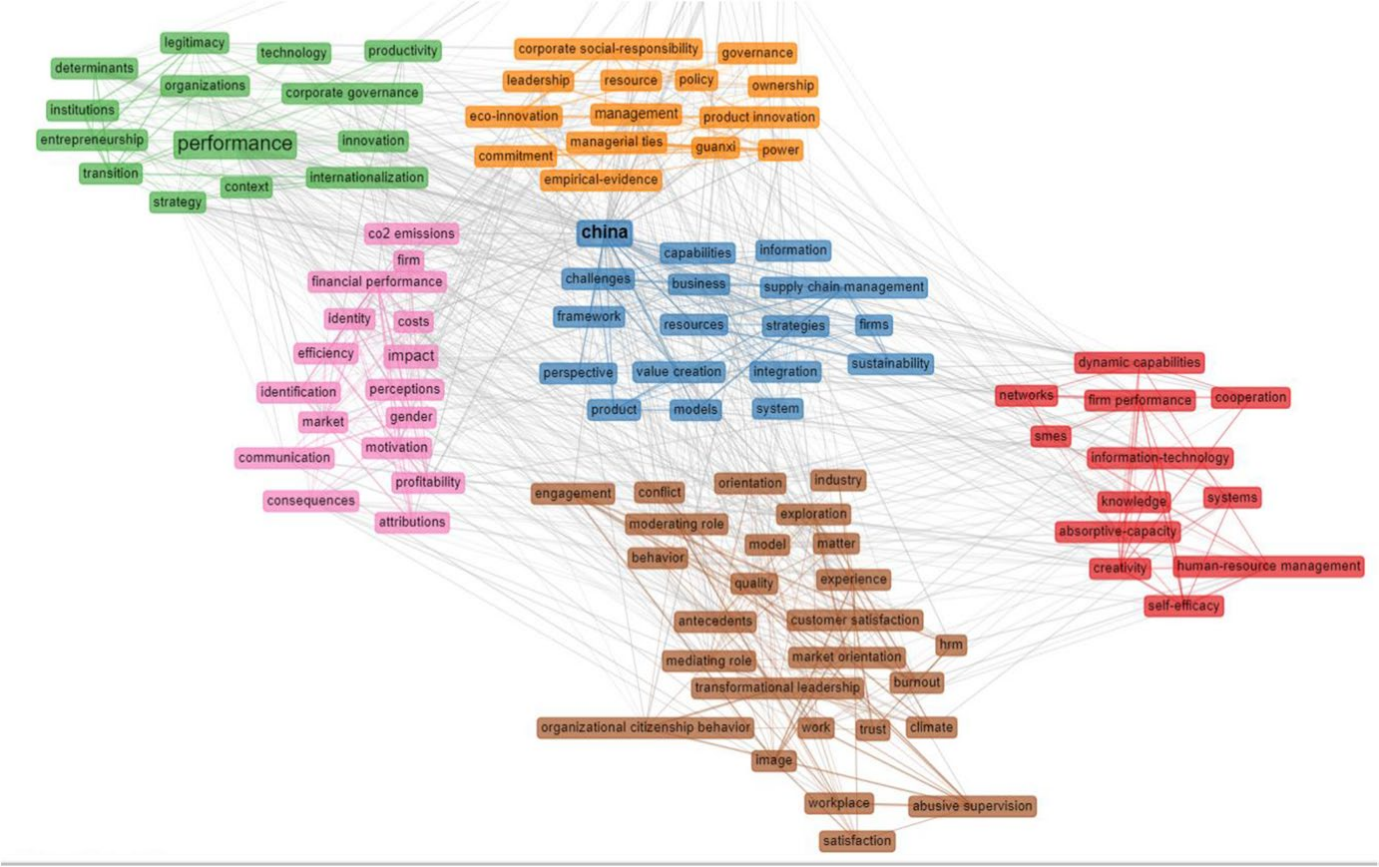
Network Map. Source: Authors’ Elaboration

The macro themes are grouped in a network that has six clusters divided by colour, as shown in Fig. 8. The clusters are connected by intense filaments that converge towards “China”, located in the centre of the map. The interweaving of the filaments expresses the transversal nature of the issues addressed and reveals the connection between the issues of business models and innovation, still under investigation.

Cluster 1 (blue group) refers to those papers dealing with technological innovation and business strategies in the Chinese context (Geissinger et al., 2020; Harun & Zainol, 2018; Liu et al., 2020; Magni et al., 2021; Permatasari & Dhewanto, 2013; Teece, 2018; Velu & Khanna, 2013; Verhoef et al., 2021).

Cluster 2 (green group) refers to the papers on innovation and performance (Broekhuizen et al., 2021; Büchi, et al., 2020; D’Amato et al., 2020; Ford & Despeisse, 2016; Geissdoerfer et al., 2016).

Cluster 3 (pink group) includes external knowledge studies (Abubakar et al., 2019; Autio et al., 2018; Bouncken & Barwinski, 2021; Scuotto et al., 2017; Visser et al., 2019).

Cluster 4 (brown group) contains the articles on business models (Breier et al., 2021; Langley et al. 2021; Verhoef et al., 2021; Keiningham et al., 2020; Ferasso et al., 2020; Li, 2020; Ghezzi & Cavallo, 2020; Kohtamäki et al., 2019; Evans et al., 2017).

Cluster 5 (red group) refers to papers focused on the innovation capabilities of firms (Langley et al. 2021; Ferasso et al., 2020; Ashrafi et al., 2019; Warner & Wäger, 2019; Teece, 2018; Rajapathirana & Hui, 2018; De Massis et al., 2018; Teece, 2014).

Cluster 6 (orange group) contains the papers on CSRV and digitization (Langley et al. 2021).

The network analysis confirmed the correlation of the topics investigated within the Asian socio-economic context. Despite the economic importance and repercussions of technological innovation linked to the industrial production of Asian countries, still few exploratory attempts have identified practical tools for the coherent integration of business models into RBMI, including the dimensions of RI. As Sun et al. confirmed (2020), among the causes, the diversity of heterogenous knowledge sources makes it difficult to identify CSRV in doing business especially looking to the emerging economies.

## Discussion of findings

Although prior studies on RBMI have tended to focus on the innovation performance and socio-environmental effects on business models, the aspect related to the responsiveness strategy adopted by firms was less understood. However, the results of the bibliometric analysis show that organizational architecture of firms has evolved in step with technological and digital transformation, enhancing the role of stakeholders in the value creation process (Broekhuizen et al., 2021; Büchi et al., 2020; Henry et al., 2020; Liu et al., 2020; Verhoef et al., 2021). The configuration of RBMI is based on strategic resource planning, incorporating innovation to include environmental, economic, and social concerns within business challenges (Stilgoe et al., 2013). Hence, RBMI requires an understanding of the plethora of stakeholders with which the company interfaces, in order to identify areas of vulnerability related to the socially oriented strategies and corrective measures of the BMI as well. In particular, innovation encourages the responsiveness of organizations with respect to meeting the needs of stakeholders (De et al., 2020), through a creative approach (Teece, 2018) inspired by the principles of inclusiveness and social well-being (Bridoux & Stoelhorst, 2016).

These values ​​express CSRV, which realizes the interconnection between corporate strategy, and ethical and social concerns (Genus & Stirling, 2018; Golob & Bartlett, 2007; Malhotra et al., 2017). Accordingly, RBMI includes the comprehensive long-term interests of innovation that consider how social, cultural, economic, and environmental aspects impact on CSRV competitiveness and the inclusion of stakeholders for the innovation process (Hadj, 2020; Stilgoe et al., 2013). Responsiveness is based on the dynamic interaction between organizations and stakeholders to contain negative externalities related to organization production (Carayannis et al., 2015; Nardi, 2021). As such, responsiveness goes beyond responsibility because it expresses the interaction between firms and the external environment manifesting itself through a contamination of “good” knowledge management practices (KMP) (Guadamillas-Gómez & Donate-Manzanares, 2011). Hence, responsiveness is connected to responsibility by a “cause and effect” relationship, which expresses a *sinallagma* among the actions “to be carried out” in response to the expectations of stakeholders, sensitive to ethical and social considerations. In this way, sustainability issues are included in corporate innovation strategies, according to the open innovation model (Long & Blok, 2018).

In fact, organizations have a dynamic connection with the community, meaning they need to plan strategies capable of mitigating economic and social purposes, adapting the business model (Langley et al., 2021) using new technologies (Keiningham et al., 2020). Several scholars have highlighted that innovating in business models also improves the ability to respond to crisis events and to react to unexpected ones (Breier et al., 2021; Kraus et al., 2020).

Organizations increasingly operate as open systems, which survive in dynamic high-risk contexts by building business models with a high degree of organizational and innovative sustainability (Carayannis et al., 2015; Kern et al., 2019; Singh & Gaur, 2020). Therefore, firms are developing dynamic adaptation strategies for technological opportunities based on the reconfiguration of the value proposition or operational framework (Ashrafi et al., 2019; D'Amato et al., 2020; Ghezzi & Cavallo, 2020; Keiningham et al., 2020; Kohtamäki et al., 2019; Li, 2020), and integrating new values ​​into the business model (Geissinger et al., 2020; Langley et al., 2021) to make the business offer more sustainable and functional (Evans et al., 2017; Ford & Despeisse, 2016; Geissdoerfer et al., 2016; Kraus et al., 2020).

Considering the challenges set by the Asean Economic Community Blueprint 2025,^1^ Association of Southeast Asian Nations (ASEAN) countries also show the need to converge towards strategic business models, deriving from the acquisition of external knowledge and the network of stakeholders, according to the paradigm of open innovation (Harun & Zainol, 2018). ASEAN countries record the highest growth rate for the intelligent exploitation of technologies, with significant effects on the economic and social development of countries (Magni et al., 2021), as well as on business models. Indeed, disruptive changes in the Asian business environment make competition around technological opportunities stronger compared to Europe or other countries (Permatasari & Dhewanto, 2013). Technologies and business dynamics in the Asian context have attempted integrating efficiency improvements into Business Model Innovation (BMI) in Asia over the last decade (Magni et al., 2021; Velu & Khanna, 2013). BMI and responsible value proposition should consider emerging market and customers’ needs, through the creation of an agile model capable of exploiting innovation adaptive capabilities of the firm (Abdulkader et al., 2020; Del Giudice et al., 2021; Foss & Saebi, 2017), integrating responsiveness into the technical infrastructure and operational processes (De Massis et al., 2018; Ferasso et al., 2020; Ghezzi & Cavallo, 2020; Rajapathirana & Hui, 2018). The focus of scholars on reflexivity and business ethic values within the Asian framework background, allows us to address the new main pillars of RBMI. RBMI is increasingly becoming a priority for firms in Asia to create competitive advantage. Strategic skills and dynamic capabilities explained by RBMI make it possible to develop business models that are durable, responsible, and adaptable to changes in the external environment in Asia (Velu & Khanna, 2013; Zhou & Li, 2007). These processes determine a relapse of knowledge towards the development of holistic systems, characterized by the sharing of information and technologies among the actors involved in the value chain (Abubakar et al., 2019; Autio et al., 2018; Foss & Saebi, 2017; Visser et al., 2019), for the construction of RI (Stilgoe et al., 2013).

In the network of relationships between the internal and external actors of the company, the knowledge of technological opportunities requires the transfer and sharing of information between individuals in a transversal manner (Bouncken & Barwinski, 2021; Scuotto et al., 2017), thus improving the responsiveness and reflexivity of the organization (Chen & Choi, 2005; Stilgoe et al., 2013). RKE activates a mechanism for involving the corporate network in the value creation process which, due to the development of dynamic capabilities, supports new innovations and knowledge exchange practices (Abdulkader et al., 2020; Nguyen et al., 2015). CSRV, inclusiveness based on Stakeholder theory, and RKE encourage the creation of strategic RBMI, based on the integration of social and ethical values ​​in the corporate strategy, nurturing the relational strength of the actors inside and outside the company and developing organizational skills capable of challenging current BMI.

By focusing on the Asian scenario, our results show the relevance of knowledge exchange by applying the lens of social exchange (Kim et al., 2017) and value co-creation (Sarma & Sun, 2017). Moreover, Permatasari and Dhewanto (2013) showed that BMI strategies focused on reconfiguring the value proposition to customers and partners practiced by Indonesian herbal cosmetics and health product companies, have fostered competitive advantage over global competitors. Innovation is also the keystone for the development of emerging economies such as India, where incumbent firms and emerging organizations are converging towards BMI by adopting new systems of product or service offerings to customers that improve performance (Velu & Khanna, 2013). Another example is in Malaysia, where the ability to innovate BM and exploit new technologies will be the basis for attracting new investments, capable of generating new wealth and increasing employment (Harrison et al., 2018).

According to a study conducted on Airbnb users from the Philippines, Indonesia and Singapore, factors such as trust, intuitiveness or convenience of the service also determine the success of BMI. This is the case of Airbnb, which has built a system based on the elimination of intermediaries, creating a direct connection between hosts and travellers (Chua et al., 2020). It is evident that responsibility is the engine for long-term persistent and sustainable BMI, favouring the development of new organizational skills, a better allocation of resources (i.e., circular BM), the responsible exchange of knowledge with the market, as well as maximizing the reactivity and reflexivity of the organization (Del Giudice et al., 2020).

### Theoretical and practical implications

Summarizing our contributions, this article provides both practical and theoretical implications leading to a better understanding of RBMI, which is emerging as a contingency mechanism that supports firms to invest responsibly and integrate sustainability into innovation processes. The first theoretical implication is the clarification of the pillars through which RBMI concerns Asian organizations, even if research on CSRV, RI, and ethical values is scant. We propose that RBMI serves as an innovative and sustainable framework that shapes the way Asian organizations forest knowledge-sharing exploration as well as disruptive technology exploitation to achieve a sustainable and competitive business model. Although the extant literature suggests that BMI is embraced by firms to balance the tensions between exploitation and exploration (Abdulkader et al., 2020; Evans et al., 2017; Foss & Saebi, 2017), we know very little about how RBMI can leverage the potential of RKE and CSRV to generate innovative dynamic capabilities for organizations. Our study bridges this theory gap by exploring the pillars of RBMI in the Asian context. Accordingly, CSRV, RI, inclusiveness, and RKE are interpreted as composite and main elements of a holistic system of RBMI.

Secondly, our study also expands the body of research around stakeholder theory and knowledge diversity for responsible innovation in socioeconomic settings (Gray et al., 2012; Nikas et al., 2017). Previous work has examined various benefits for stakeholder theory approach, such as satisfying the needs of the whole business environment (Freeman et al., 2010; Bridoux & Stoelhorst, 2016). Our study adds further insights to this stream of the literature by proposing the inclusiveness aspects of third parties in Asia. Finally, our study expands the themes of knowledge exchange and sustainability in Asia. We offer a theoretical and scientific approach to analysing the dynamic capabilities formed by RBMI and include them within an innovative and sustainable theoretical framework. We also systematically describe the relationships between CSRV, RI, and RKE by following the sustainability perspective in Asia.

The paper also offers managerial and practical contributions. We highlighted the role of RBMI for managers and practitioners to cope with new sustainability and responsibility challenges in Asian organizations. Our results offer managers a systematic key to interpreting and approaching sustainability challenges. Above all, they indicate a path for managers regarding transformational dynamic capabilities activated by CSRV, RI and RKE to reimagine RBMI and create new value. Ethics corporate dynamics capabilities help knowledge-intensive organizations to gain a competitive advantage by creating responsible and stakeholder-oriented interactive relationships within prosocial business environments (Singh & Gaur, 2020). Therefore, managers can develop a new sustainable and responsible mindset to operate among increasingly disruptive transformation, identifying aspects of vulnerability and the potential of the business with respect to environmental and social well-being issues (Shirahada & Zhang, 2021). In this respect, our paper provides several elements to be taken into consideration in an Asian scenario involving responsiveness and inclusiveness, in order to map the ecosystem of stakeholders connected to the firm.

While this research provides initial insights into the architecture of RBMI in Asian organizations, the relational approach towards CSRV should be extended to all companies to understand how to create more responsible and shared value for all stakeholders through RKE. This approach favours the materiality analysis of innovation scenarios, exploring trends related to business externalities and knowledge spillovers to integrate action strategy that contributes to remarkable sustainable growth.

## Concluding remarks

This paper offers a comprehensive review of innovation in the business model, reconstructing the RBMI journey in the context of Asian organizations through the lens of RI.

Considering the challenges posed by the dynamic nature of society and markets, organizations today are building a CSRV that incorporates ethical values ​​and sustainability into business strategy through open dialogue with stakeholders (Genus & Stirling, 2018; Golob & Bartlett, 2007), in order to favour RI. Indeed, RI involves corporate ethics, as it expresses the objective of combining the needs for profit with the protection of the interests of all stakeholders according to the principles of (i) inclusion (Malhotra et al., 2017); (ii) anticipation (Wickson & Carew, 2014); (iii) reactivity (Stilgoe et al., 2013); and (iv) reflexivity (Chen & Choi, 2005).

This paper examined the use of RBMI in the Asian socio-economic context, as the challenges faced by ASEAN businesses are of particular interest both in terms of helping local businesses and enriching the global academic discourse (Meyer, 2006; White, 2002). The interest in the Asian context arises from the peculiarities of the institutional context as well as from the organizational principles that characterize ASEAN companies. The organizational forms that characterize Asian BMs have evolved hand in hand with the institutional environment, according to adaptive modalities that are constantly evolving (Carney & Gedajlovic, 2002).

The results revealed the need to seize technological opportunities proactively, addressing the social and environmental threats related to business. However, the adoption of RBMI requires strong dynamic skills, linked to the development of adaptative strategies in response to technological and market opportunities. Based on the integration of social and ethical values ​​into corporate strategy, RBMI encourages the construction of a network between internal and external actors of the company, thanks to the exploration of knowledge sharing practices and the exploitation of new technologies (Ali et al., 2019; Del Giudice et al., 2021). Inside the organization, RBMI turns into dynamic capabilities supporting the decision making by employees for enhancing organizational performance on the shopfloor (Singh, Vrontis, et al., 2021).

Although ASEAN countries have also expressed the need to converge towards strategic BMs, deriving from the acquisition of external knowledge and the network of stakeholders, according to the paradigm of open innovation (Harun & Zainol, 2018), the literature on the field seems more concerned with productivity issues and the sustainability goals embedded in the RBMI.

Certainly, the economies of these countries can contribute significantly to technological development because they have a strong inclination for innovation. However, RI requires the modernization of BM, in terms of efficiency, agility and scalability, but also in terms of business responsiveness for the conscious and inclusive use of innovation. ASEAN countries record the highest growth rate for the intelligent exploitation of technologies, yet the literature does not seem to have identified RBMI as a tool that can strengthen business ethics, as well as combining profit objectives and social issues. Therefore, while innovation creates new opportunities for business by improving the product and service offer, it alone is not enough to develop CSRV. It will be necessary to adopt an integrated strategy, founded on ethical and sustainable values, which supports the involvement of stakeholders towards the creation of RBMI.

### Limitations and future research

The paper is affected by specific limitations related to the SRL we attempted to use. Firstly, the methodology is limited to subjects such as: business, technology management, and sustainability. In our work, we do not give any consideration to other scientific approaches in the Asia scenario, i.e., the impact of technology on citizens’ well-being or the development of smart cities. Secondly, in line with the main purpose of this paper, our SRL is affected by research biases in confirming our main hypotheses.

Despite these limitations, the paper stresses the need to develop much more focused research tohus improving links between RI, CSRV, and RKE especially within the Asian context. By focusing on Asia, future research and innovative frameworks would make it possible to further advance knowledge on RBMI. Especially for knowledge-intensive sectors, future research should explore RKE’s mediating role for RBMI through free innovation, as they could add further insights to CSRV strategies. More in-depth future studies should monitor the responsiveness initiatives of companies through the implementation of free innovation, empirically analysing the relationship between RKE and stakeholder engagement from a CSRV perspective.

## Footnotes

1 https://asean.org/asean-economic-community/
